# Antioxidant Status and Liver Function of Young Turkeys Receiving a Diet with Full-Fat Insect Meal from *Hermetia illucens*

**DOI:** 10.3390/ani10081339

**Published:** 2020-08-03

**Authors:** Katarzyna Ognik, Krzysztof Kozłowski, Anna Stępniowska, Piotr Listos, Damian Józefiak, Zenon Zduńczyk, Jan Jankowski

**Affiliations:** 1 Biochemistry and Toxicology, Faculty of Animal Science and Bioeconomy, University of Life Sciences in Lublin, Akademicka 13, 20-950 Lublin, Poland; anna.stepniowska@up.lublin.pl; 2 Department of Poultry Science, Faculty of Animal Bioengineering, University of Warmia and Mazury in Olsztyn, Oczapowskiego 5, 10-719 Olsztyn, Poland; kristof@uwm.edu.pl (K.K.); janj@uwm.edu.pl (J.J.); 3 Department of Pathological Anatomy, Faculty of Veterinary Medicine, University of Life Sciences in Lublin, Akademicka 13, 20-950 Lublin, Poland; piotr.listos@up.lublin.pl; 4 Department of Animal Nutrition and Feed Management, Animal Sciences, Poznań University of Life Sciences, Wołyńska 33, 60-637 Poznań, Poland; damjo@up.poznan.pl; 5 Institute of Animal Reproduction and Food Research, Polish Academy of Sciences, 10-748 Olsztyn, Poland; z.zdunczyk@pan.olsztyn.pl

**Keywords:** turkey, insect meal, biochemical parameters, redox status, growth performance

## Abstract

**Simple Summary:**

Insects can be used as an alternative source of protein and fat in poultry feed. To date, in most research, the administration of invertebrates as a replacement for soybean meal in chicken diets has produced satisfactory results. We hypothesized that full-fat insect meal from *Hermetia illucens* (HI) larvae can also be an acceptable source of protein and energy in the diet of young turkeys, and at the same time can improve their antioxidant status and metabolism. Our research showed that the level of HI meal in the diet of turkeys should not exceed 5%. The use of a higher level of HI than 5% in the diet of young turkeys has a negative effect on lipid metabolism, lipid oxidation and fat deposition in the liver.

**Abstract:**

We hypothesized that full-fat insect meal from *Hermetia illucens* (HI) larvae can be an acceptable source of protein and energy in the diet of young turkeys, in an amount adapted to the nutritional needs of these birds, and at the same time can improve their antioxidant status and metabolism. The turkeys were fed a control diet (HI_0_) without the insect meal, and three diets with increasing HI content of 5%, 10% and 15% (treatments HI_5_, HI_10_ and HI_15_, respectively). The use of 10% or 15% HI in the diet of young turkeys, while beneficially raising levels of P, Fe and Hb, has a negative effect on lipid metabolism, increasing TC levels, lipid oxidation, and fat deposition in the liver. The inclusion of 5% HI in the diet of young turkeys has no adverse effect on the lipid status and histology of the liver, but it does not improve antioxidant status. To conclude, the level of HI meal in the diet of turkeys should not exceed 5%. However, as similar studies on turkeys have not yet been published, overly general conclusions should not be drawn from the results of the present study, and further research is necessary.

## 1. Introduction

Numerous studies have been undertaken in recent years to determine the possibility of using meal or oil from insect larvae in the diet of poultry [1,2,3]. According to [2], the use of insect products in the diets of birds could reduce the negative impact of poultry production on the environment. The authors point out that the production of feed from insects requires a significantly smaller soil surface area, and less water, than the plants conventionally used as protein sources. In addition, insects emit less ammonia and greenhouse gases into the environment during their development. Data published by Dossey et al. [4] indicate that less than 1 tonne of soy protein per year can be produced from 1 ha of arable land, while up to 150 tonnes of insect protein can be produced from the same area. Insect larvae can also be used as a waste bioreactor [5].

Many authors report that insect products have a high nutritional value, so they can exert a beneficial effect on growth, nutrient digestibility and health in poultry [2,6,7,8]. Black soldier fly (*Hermetia illucens*, HI) larvae are a rich source of fat (7–39% dry matter—DM) and protein (37–63% DM), with a better amino acid profile than soybean meal [9,10]. The fatty acid profile of HI larvae depends on the fatty acid composition of the medium on which they are reared. Insect larvae are rich in lauric acid (20–40% of all lipids), palmitic acid (11–16% of all lipids) and oleic acid (12–32% of all lipids) [2].

HI larvae also contain chitin in the amount of 5.9% to 8.7%. In addition to chitin, antimicrobial peptides (AMPs) also have a stimulating effect on the immune system [11,12]. AMPs are small cationic peptides that show a broad spectrum of activity against bacteria, fungi and viruses [12,13,14,15]. HI larvae are also rich in mineral elements, especially Ca (5–8% DM) and P (0.6–1.5% DM) (10). According to Secci et al. [16], insect meal is a rich source of low-molecular-weight antioxidants, including carotenoids and tocopherols, which can increase the body’s antioxidant potential.

Many studies on chickens have shown the beneficial effects of the use of insect larvae products on the immune system, the composition of the gastrointestinal tract (GIT) microbiota, and production results, owing to their high content of biologically active substances [17,18,19,20,21]. However, the literature also includes poultry studies that do not confirm their immunostimulatory and antioxidant properties, or their beneficial effects on metabolism or growth performance [3,9]. The discrepancies in results may be due to the fact that the experiments use different types of insects, in different forms (meal or oil) and as different proportions of the diet. However, despite the growing interest in research into the potential use of HI as a dietary component to improve the growth performance, immunity and antioxidant status of birds, such information is still lacking with respect to turkeys, a species requiring a high-protein diet, especially in the first few weeks of life.

Therefore, we hypothesized that full-fat insect meal from *Hermetia illucens* larvae can be an acceptable source of protein and energy in the diet of young turkeys, in an amount adapted to the nutritional needs of these birds, and at the same time can improve their antioxidant status and metabolism.

## 2. Material and Methods

### 2.1. Birds and Housing

The experiment was carried out in a poultry house at the experimental facilities of the Department of Poultry Science, University of Warmia and Mazury in Olsztyn, Poland. A total of 432 one-day-old female Hybrid Converter turkeys were randomly assigned to four treatment groups, with 12 replicates of nine birds each. The birds were kept in cages (0.5 × 1 m), and all birds had free access to feed and water. Each cage was equipped with nipple drinkers and a feeder that was manually filled daily. The temperature and lighting programs were in compliance with the requirements of Hybrid Turkeys [22]. The protocol for the study was approved by the Local Ethics Committee, and the animals were cared for under guidelines consistent with those laid down by EU Directive 2010/63/EU.

### 2.2. Experimental Design and Diets

The turkeys were fed a control diet (HI_0_) without the insect meal and three diets with increasing HI contents of 5%, 10% and 15% (treatments HI_5_, HI_10_ and HI_15_, respectively). Full-fat meal from *Hermetia illucens* larvae was obtained from a commercial source (HiProMine S.A., Robakowo, Poland). The meal was air-dried in an oven (SLN 240, POL–EKO Aparatura, Poland) for 24 h at 50 °C and finely ground (Zelmer motor blocked power 1900 w, Rzeszów, Poland) to obtain full-fat meals. All diets were prepared at the agrocentrum sp. z o.o. feed mill, according to the nutrient requirements of young turkeys [22]. The composition of full-fat *Hermetia illucens* meal is given in Table 1. The basal diet, whose composition is given in Table 2.

### 2.3. Sample Collection

At 28 days of age, blood samples were taken from 12 turkeys from each group. Then the same birds were tagged. The turkeys were killed at a slaughterhouse. The birds (without being transported) were electrically stunned (400 mA, 350 Hz), hung on a shackle line, and exsanguinated by a unilateral neck cut severing the right carotid artery and jugular vein. After a 3 min bleeding period, the birds were scalded at 61 °C for 60 s, defeathered in a rotary drum picker for 25 s, and manually eviscerated. Liver samples were collected for histological examination.

### 2.4. Chemical Analyses

The plasma content of total cholesterol (TC), total protein (TP), glucose (GLU), Ca, P, Mg, Fe, Cu and Zn, as well as the activity of aspartate aminotransferase (AST), were measured using an automatic biochemical analyzer (Plasma Diagnostic Instruments Horiba, Kyoto, Japan). Malondialdehyde (MDA) was determined in the blood and liver as a marker of oxidative stress, using kits produced by Cell Biolabs, Inc. (San Diego, CA, USA). A diagnostic kit manufactured by Oxis International, Inc. (Portland, OR, USA) was used to determine superoxide dismutase (SOD) and catalase activity (CAT) in plasma and liver homogenates. Glutathione (GSH + GSSG) concentrations were determined using a Total Glutathione Assay (Cell Biolabs, Inc., San Diego, CA, USA).

### 2.5. Histological Examination of Tissue Samples

Samples of the liver were cut in two lengthwise, and fixed for 24 h in 5% formalin, pH 7.2. Within 24 h the fixed tissue fragments were passed through increasing concentrations of alcohol solutions, acetone and xylene into paraffin blocks in a tissue processor (Leica TP-20; Leica Biosystems, Buffalo Grove, IL, USA). Paraffin-embedded microscope sections 5 μm thick were stained with haematoxylin and eosin (HE staining). Morphometric evaluation of the tissues was carried out using a computer-assisted microscopic image analysis system. The system includes a light microscope (Nikon Eclipse E600; Medtech-Supplies, Hertfordshire, United Kingdom) with a digital camera (Nikon DS-Fi1; Nicon Corporation, Tokyo, Japan) and a PC with image analysis software (NIS-Elements BR-2.20, Laboratory Imaging, Praha, Czech Republic).

### 2.6. Statistical Analysis

The Statistica software package version 13.1 was used to determine whether variables differed between treatment groups. When the ANOVA indicated significant treatment effects, means were separated using Tukey’s multiple range test. The results are presented in the tables as mean values with pooled standard errors. Data were checked for normal distribution before the statistical analysis was performed. Differences were considered significant at *p* ≤ 0.05.

## 3. Results

During the four weeks of the experiment, there was a linear decrease in feed conversion ratio—FCR (*p* = 0.011), with the lowest value recorded in the HI_15_ treatment. A linear upward trend in final body weight—BW was observed (*p* = 0.056). The liveability was the same in all experimental groups (approximately 99%; Table 3).

The inclusion of 10% and 15% HI in the compound feed resulted in an increase in blood levels of Hb (*p* = 0.001), GLU, TC and AST (*p* < 0.001, all). The Hb level showed a linear and quadratic response (*p* = 0.001 and *p* = 0.061, respectively), with the highest values in the HI_10_ and HI_15_ treatments. The GLU level showed a linear response (*p* < 0.001), with the highest value in the HI_15_ treatment. The highest TC levels were observed in the HI_10_ and HI_15_ treatments, with linear and cubic responses (*p* < 0.001, both) in the other treatments. AST activity showed a linear and quadratic response (*p* < 0.001 and *p* = 0.002, respectively), with higher values in the HI_10_ and HI_15_ treatments compared to HI_0_ and HI_5_. The TP level in the blood plasma showed a cubic response (*p* = 0.014), with the highest value in the HI_5_ treatment and the lowest in HI_10_ and HI_15_. The inclusion of 15% HI in the compound feed reduced plasma SOD activity in the turkeys relative to group H_I0_ (*p* = 0.027). Increasing the proportion of HI in the diet resulted in a linear decrease in SOD activity (*p* = 0.004; Table 4).

The inclusion of 10% and 15% HI in the compound feed resulted in an increase in plasma Fe and Zn levels (*p* < 0.001, both). The Fe level showed a linear and cubic response (*p* < 0.001 and *p* = 0.048, respectively), with the highest values obtained in the HI_10_ and HI_15_ treatments. The Zn level showed a linear response (*p* < 0.001), with the highest values in the HI_10_ and HI_15_ treatments. The inclusion of 15% HI in the compound feed resulted in an increase in the plasma *p* level (*p* = 0.006) in the turkeys. The *p* level showed a linear response (*p* = 0.001), with the highest value in the HI_15_ treatment (Table 5).

The inclusion of HI in the feed, irrespective of the amount, caused no significant differences in CAT activity relative to group HI_0_, but the highest CAT activity of this enzyme was noted in group HI_10_, with a difference relative to group HI_5_ (*p* = 0.049). A cubic response (*p* = 0.031) was also noted for CAT activity. In the HI_10_ treatment, there was a downward tendency in SOD activity (*p* = 0.054) and an upward tendency in the MDA level (*p* = 0.072) compared to the HI_0_ treatment. The use of 15% HI decreased the plasma level of GSH + GSSG in the turkeys (*p* = 0.002). The GSH + GSSG level showed a linear response (*p* < 0.001), with the lowest value in the HI_15_ treatment (Table 6).

**Figure 1:**
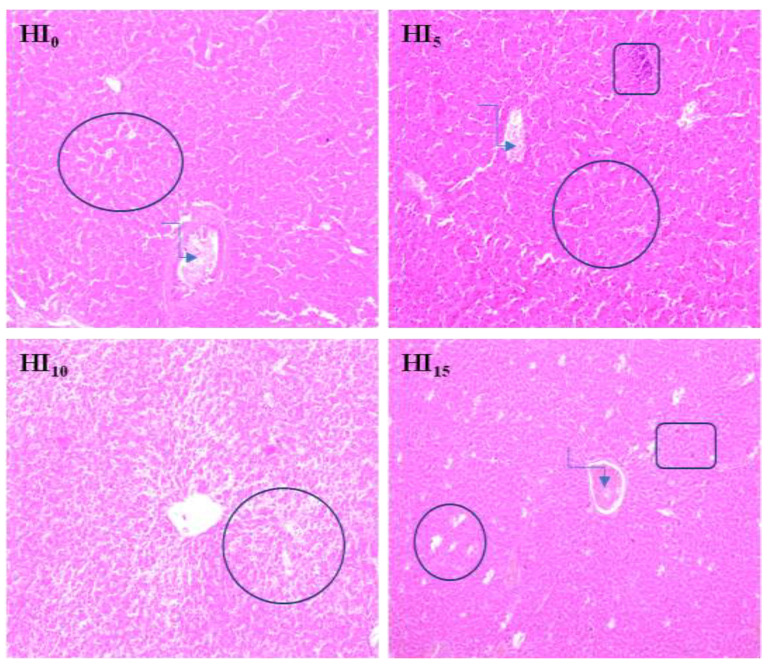
Morphological effects of different HI levels in feed on turkeys liver (magnification 10×). Treatments: HI_0_–group without HI in diet; HI_5_—group with 5% HI in diet; HI_10_—group with 10% HI in diet; HI_15_—group with 15% HI in diet. 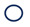 foci of fatty degeneration; 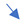 blood stasis in the central vein; 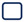 local infiltration of mononuclear cells

The histological examination of the livers of turkeys from the H_I0_ treatment revealed no pathological changes. The histological structure was normal, with small physiological foci of fatty degeneration. The histological structures of the livers of turkeys from the H_5_ group were normal, with numerous physiological foci of fatty degeneration, central venous stasis, and isolated mononuclear cell infiltrates. In contrast, in group HI_10_, the histological examination of the liver showed a significant degree of generalized pathological fatty degeneration, with characteristics of steatosis. Similarly, in the livers of turkeys from the HI_15_ treatment, single, severe foci of fatty degeneration were found, accompanied by central venous stasis and numerous (minor) infiltrates of mononuclear cells (Figure 1).

## 4. Discussion

To the best of the authors’ knowledge, this study is the first to have tested HI larva meal in the diets of young turkeys. According to Dabbou et al. [9], a 10% share of HI meal in place of soybean meal is suitable as a feed component in the initial period of chicken feeding. However, our research showed improvement in the rearing results (FCR only) of young turkeys only when the share of HI in the diet was increased to 15%. Oluokun [24] and Loponte et al. [7] noted improvements in the growth performance of chickens and partridges receiving HI as a diet component replacing 25% or 50% of soybean meal. Khan et al. [25] reported that the substitution of soya meal with maggot meal significantly decreased feed intake while increasing body weight. The literature to date provides very little data on the effect of the use of insect meal on turkey production results. A study was recently conducted on turkeys receiving a diet with insect larvae oil (not meal). The authors found that the partial or complete replacement of soybean oil with HI oil did not affect rearing results [3].

The total protein concentration in the blood is an important biomarker of the physiological state of animals, which is affected by the quantity and quality of protein in their feed. In our study, the higher plasma TP level in the turkeys receiving a diet in which soybean meal was replaced with 5% HI meal indicates that this diet had a beneficial effect on protein metabolism. Research into chickens receiving a diet with 25%, 50%, 75% or 100% HI in place of fishmeal observed no effects on the plasma levels of TP [26]. Similarly, Gariglio et al. [2] reported that replacing soybean meal with HI in the amount of 3–9% did not affect the plasma TP level in ducks.

In the present study, the inclusion of 10% or 15% HI meal in the diet of young turkeys resulted in an increase in the plasma content of phosphorus and zinc. An increased plasma level of phosphorus in chickens receiving a 5%, 10% or 15% share of HI meal in the diet has been reported by Dabbou et al. [9]. The higher phosphorus levels in the plasma of birds receiving a diet with HI may be associated with the higher phosphorus availability from HI larva meal, as compared to plant components [27]. The phosphorus content of HI larvae has been shown to be closely correlated with the phosphorus content of the medium used to rear the larvae [28,29]. There are also reports showing that replacing soybean meal with HI has no effect on the P level in the plasma of birds [2]. Data presented by Dierenfeld and King [30] show that HI larvae are a rich source of Zn, which they probably take up from the substrate provided to them. Thus, as in the case of P, the increased plasma Zn content in the turkeys may be due to greater access to this element.

In our study, the inclusion of 10% and 15% HI in the diets of young turkeys increased Fe and Hb levels. Iron is an element essential for the production of erythrocytes. It is a component of haem contained in haemoglobin, i.e., the protein in erythrocytes [9]. The level of HI meal in the diet of birds can unquestionably have varied effects on the plasma content of Fe, and thus on haemoglobin production. Gariglio et al. [2] did not find elevated plasma levels of iron in ducks that received 3% to 9% HI in their diet. Similarly, Marono et al. [18], in an experiment on laying hens receiving a diet in which soybean meal was completely replaced with HI meal, noted no effect on Fe and Hb levels. Bovera et al. [31] also found no increase in the Hb level in the blood of broiler chickens receiving a diet in which soybean meal was completely replaced with *Tenebrio molitor* meal. Research by Schiavone et al. [32] showed that 50% or 100% replacement of soybean oil with HI fat also had no effect on the Fe levels in the blood, or on erythropoiesis, in chickens. The literature’s data indicate that chemical chelation of Fe by dietary fiber in HI larvae may take place in the gastrointestinal tract, which may reduce the bioavailability of Fe if the levels of insect larvae meal in the diet are too high [30]. The contrasting results of our study, i.e., an increase in the level of Fe and Hb, may be due to the use of much lower levels of HI meal in the turkey diet. The data presented by Dierenfeld and King [30] indicate that HI larvae are a rich source of Fe (about 370 mg/kg). It is possible that Fe, like other minerals, was chemically bound in the sclerotinized cuticle [30]. In our study, the use of 5% HI in the diet of young turkeys did not affect the plasma level of GLU, but increasing the share of HI to 10% and 15% resulted in an increase in this parameter. A study of laying hens receiving a diet in which soybean meal was 100% replaced with HI meal found no effect on the plasma level of GLU [18]. Long-term increased GLU levels in the body promote the induction of glycogenesis and thus glycogen deposition in the hepatocytes [2,31].

According to some authors [18,31,33], when HI meal is used in the diet of birds, a favorable decrease in plasma TC levels can be expected, because it is assumed that chitin from the insect meal can react with bile acids and free fatty acids. Marono et al. [18] demonstrated a decrease in plasma TC levels in layers receiving a diet in which soybean meal was 100% replaced with HI meal. In contrast, a study by Schavione et al. [34] on chickens receiving a diet in which soybean oil was replaced with 50% or 100% HI fat found no effect of the experimental factor on TC levels. It is also likely that the use of high levels of both HI meal and HI oil in the diet of birds can adversely affect their lipid profile. Oil from insect larvae contains four times the level of saturated fatty acids that are in soybean oil [35]. Data presented by numerous authors show that the fatty acid profile of HI larvae oil has a high proportion of medium-chain fatty acids (MCFA), including lauric acid (C12:0), and esters, which constitute 21% to 49.3% of the entire profile [26,36,37]. In our research, the inclusion of 5% HI in the compound feed of young turkeys had no effect on plasma TC levels, but higher proportions of the meal (10% or 15%) resulted in an unfavorable increase in this parameter. The mobilization of lipids from the liver to peripheral tissues is generally associated with the availability of transport lipoproteins [38]. In our study, the use of 10% or 15% HI in the diet of young turkeys also caused adverse histological changes in the liver, manifested as fatty degeneration. Severe liver damage was found in an experiment on ducks that received from 3% to 9% HI meal in the diet [2]. There are also studies on chickens receiving HI or TM meal in the diet [6,9,20,39], in which histological examination of the liver found no damage. Elia et al. (2018) noted pathological fatty changes in the livers of rainbow trout receiving 25% or 50% HI meal in the diet in place of fishmeal. In an experiment on clownfish, Vargas-Abúndez et al. [40] replaced fishmeal with 25%, 50% or 75% HI meal and found no pathological changes in the liver. Although studies in chickens have not found any negative changes in the liver resulting from the use of HI in the diet, the steatosis of this organ observed in our study may be due to the fact that turkeys may have a greater tendency to accumulate lipids than chickens, which would be worth establishing in subsequent research. Chartrin et al. [41] and Hérault et al. [42] report that fat deposition in the liver may vary even within a single bird species, e.g., in Muscovy ducks and Pekin ducks.

In our study, steatosis of the liver in young turkeys receiving a diet with 10% and 15% HI was also reflected by increased activity of AST in the blood plasma. Fat deposition in the liver can lead to damage of the hepatocytes. Increased AST activity is often noted even in the case of minor hepatocyte damage, as well as in chronic liver disease [43]. Increased AST activity has also been reported by Chaklader et al. [44], who used HI meal in the diet of *Vibrio harveyi*. In contrast, Marono et al. [18] found that replacing soybean meal with HI meal in laying hens had no effect on AST activity. Schiavone et al. [34] showed that replacing soybean oil with 50% or 100% HI fat also did not affect AST or ALT activity in chicken plasma.

Pathological changes in the liver are also a consequence of lipid peroxidation reactions, which generate oxidative stress in cells. Malondialdehyde is an end product of lipid peroxidation, and at the same time a marker of the intensity of this process. Few researchers have analyzed the response of the antioxidant system of birds to the use of meal or oil from insect larvae in the diet. The few available data indicate that the inclusion of HI meal in the diet of ducks does not induce oxidative reactions in tissues [2]. Moreover, the authors of the study report that HI meal in the diet of ducks stimulates their antioxidant system. In contrast, our research shows that just 10% HI in the diet of young turkeys intensified oxidative reactions in the liver, which resulted in an increase in MDA levels and stimulation of the antioxidant enzyme SOD. A study on rainbow trout receiving 25% or 50% HI meal in the diet in place of fishmeal revealed steatosis of the liver, but did not show an increase in lipid oxidation or changes in the activity of antioxidant enzymes SOD and CAT. The authors of the study noted a favorable increase in GSH + GSSG levels in the groups receiving HI [45].

## 5. Conclusions

The use of 10% or 15% HI in the diet of young turkeys, while beneficially raising levels of P, Fe and Hb, has a negative effect on lipid metabolism, increasing TC levels and lipid oxidation. Additionally, the use of 10% or 15% HI in the diet of turkeys has negative effects on liver metabolism, by increasing AST activity and fat deposition in the liver.

The inclusion of 5% HI in the diet of young turkeys has no adverse effect on the lipid status and histology of the liver, but it does not improve antioxidant status.

To conclude, the level of HI meal in the diet of turkeys should not exceed 5%. However, as similar studies on turkeys have not yet been published, overly general conclusions should not be drawn from the results of the present study, and further research is necessary.

## Figures and Tables

**Table 1:** Analysis of the chemical composition of full-fat *Hermetia illucens* meal (HI).

Item	HI
Dry matter, g/100 g	97.50
Crude protein, g/100 g	40.4
Ether extract, g/100 g	33.5
Calcium, g/100 g	1.36
Phosphorus, g/100 g	0.79
Zinc, mg/kg	146
Copper, mg/kg	11
Chitin, g/100 g	8.0
Fatty acid profile, g/100 g	
C8:0 Caprylic acid	0.15
C12:0 Lauric acid	21.0
C14:0 Myristic acid	2.65
C14:1 Miristoleic acid	0.08
C16:0 Palmitic acid	3.54
C16:1n7 Palmitoleic acid	1.05
C18:0 Stearic acid	0.45
C18:1n0 Oleic acid	2.71
C18:2n6 Linoleic acid	1.73
C18:3n3 α-Linoleic acid	0.15
C20:0 Arachidic acid	0.08
C20:4n-6 Arachidonic acid	0.03
C20:5n-3 Eicosapentaenoic acid	0.03
C22:6n-3 Docosapentaenoic acid	0.03
SAFA	18.73
MUFA	3.84
PUFA	1.97
Total Omega-3	0.15

SAFA: Short-chain fatty acids; MUFA: Monounsaturated fatty acids; PUFA: Polyunsaturated fatty acid.

**Table 2:** Composition and nutrient density of the experimental diets, g/100g.

	Diet ^1^			
	HI_0_	HI_5_	HI_10_	HI_15_
Component				
Wheat	43.51	44.80	46.09	47.41
Soybean meal	42.94	38.47	33.99	29.50
Black soldier fly meal	-	5.00	10.00	15.00
Rapeseed meal	3.00	3.00	3.00	3.00
Soybean oil	4.88	3.27	1.66	0.05
Salt	0.30	0.30	0.29	0.28
Limestone	1.79	1.71	1.64	1.56
MCP	2.29	2.14	2.00	1.86
Choline chloride	0.10	0.10	0.10	0.10
DL-Methionine	0.33	0.34	0.35	0.36
L-Lysine	0.49	0.50	0.51	0.51
L-Threonine	0.12	0.12	0.12	0.12
Vitamins + trace minerals ^2^	0.25	0.25	0.25	0.25
Calculated nutrient density ^3^				
ME (kcal/kg)	2850	2850	2850	2850
Crude protein	27.50	27.50	27.50	27.50
Crude fibre	3.11	3.16	3.21	3.26
Lysine	1.80	1.80	1.80	1.80
Methionine	0.71	0.72	0.73	0.75
Met. + Cys.	1.17	1.17	1.17	1.17
Threonine	1.10	1.10	1.10	1.10
Calcium	1.30	1.30	1.30	1.30
Available phosphorus	0.70	0.70	0.70	0.70
Analysed nutrients				
Crude protein	28.26	27.92	27.14	28.47
Crude fat	6.84	6.17	5.63	5.29
Dry matter	90.54	90.33	90.08	90.51
Crude ash	7.35	7.24	7.22	7.39
MJ ME/kg	17.68	17.70	17.54	17.66

^1^ HI_0_—group without HI in diet; HI_5_—group with 5% HI in diet; HI_10_—group with 10% HI in diet; HI_15_—group with 15% HI in diet; ^2^ Provided per kilogram of diet: 12.500 IU vitamin A; 5000 IU vitamin D_3_; 100 IU vitamin E; 4.0 mg vitamin K; 4.5 mg vitamin B1; 15 mg vitamin B_2_; 5 mg vitamin B_6_; 0.04 mg vitamin B_12_; 110 mg nicotinic acid; 28 mg pantothenic acid; 3.5 mg folic acid; 0.375 mg biotin; 80 mg iron; 25 mg copper; 160 mg manganese; 160 mg zinc; 2.5 mg iodine; 0.3 mg selenium; 0.36 g calcium ^3^ Calculated according to Polish Feedstuff Analysis Tables [23]. MCP: Metabolizable Crude protein; ME: Metabolizable Energy.

**Table 3:** Growth performance of turkeys (0–28 days).

	Initial BW kg	Final BW kg	FCR kg/kg	Liveability %
Group ^1^				
HI_0_	0.059	1.104	1.642 ^b^	99.07
HI_5_	0.059	1.121	1.604 ^ab^	99.07
HI_10_	0.059	1.129	1.609 ^ab^	99.07
HI_15_	0.059	1.149	1.577 ^a^	99.07
SEM	0.0001	0.008	0.008	-
*p*-value				
Group	-	0.074	0.042	-
Linear	-	0.056	0.011	-
Quadratic	-	0.936	0.877	-
Cubic	-	0.775	0.272	-

^a, b^ mean values within column with unlike superscript letters were shown to be significantly different (*p* < 0.05). ^1^ HI_0_—group without HI in diet; HI_5_—group with 5% HI in diet; HI_10_—group with 10% HI in diet; HI_15_—group with 15% HI in diet. BW—body weight; FCR—feed conversion ratio.

**Table 4:** Haematological and biochemical blood parameters of turkeys.

	Hb g/L	GLU mmol/L	TP g/L	TC mmol/L	MDA µmol/mL	AST U/L	SOD U/mL
Group ^1^							
HI_0_	24.22 ^b^	17.63 ^c^	22.31 ^ab^	2.137 ^b^	1.033	159.8 ^c^	36.75 ^a^
HI_5_	26.21 ^ab^	20.31 ^bc^	23.65 ^a^	2.145 ^b^	1.034	158.4 ^c^	36.65 ^ab^
HI_10_	27.96 ^a^	22.11 ^ab^	21.88 ^b^	3.443 ^a^	0.999	185.3 ^b^	34.92 ^ab^
HI_15_	27.47 ^a^	23.74 ^a^	21.91 ^b^	3.038 ^a^	0.986	223.7 ^a^	33.71 ^b^
SEM total	0.366	0.482	0.231	0.102	0.016	4.478	0.426
*p*-Value:							
Group	0.001	<0.001	0.018	<0.001	0.633	<0.001	0.027
Linear	0.001	<0.001	0.135	<0.001	0.223	<0.001	0.004
Quadratic	0.061	0.517	0.138	0.169	0.829	0.002	0.497
Cubic	0.496	0.848	0.014	<0.001	0.691	0.539	0.533

^a, b, c^ mean values within column with unlike superscript letters were shown to be significantly different (*p* < 0.05). ^1^ HI_0_—group without HI in diet; HI_5_—group with 5% HI in diet; HI_10_—group with 10% HI in diet; HI_15_—group with 15% HI in diet. Hb-haemoglobin; GLU—glucose; TP—total protein; TC—total cholesterol; MDA—malondialdehyde; AST—aspartate aminotransferase; SOD—superoxide dismutase.

**Table 5:** Minerals content in the blood of turkeys.

	Ca mmol/L	P mmol/L	Mg mmol/L	Fe µmol/L	Cu µmol/L	Zn µmol/L
Group ^1^						
HI_0_	2.957	1.400 ^b^	3.196	11.53 ^b^	50.26	58.08 ^b^
HI_5_	3.082	1.466 ^ab^	3.119	11.12 ^b^	50.70	64.05 ^b^
HI_10_	2.999	1.464 ^ab^	3.350	14.93 ^a^	51.41	75.56 ^a^
HI_15_	3.007	1.535 ^a^	2.676	15.67 ^a^	53.05	75.27 ^a^
SEM total	0.028	0.014	0.101	0.469	0.902	1.722
*p*-Value:						
Group	0.475	0.006	0.102	<0.001	0.720	<0.001
Linear	0.792	0.001	0.136	<0.001	0.273	<0.001
Quadratic	0.303	0.919	0.134	0.480	0.744	0.294
Cubic	0.244	0.233	0.173	0.048	0.934	0.194

^a, b^ mean values within column with unlike superscript letters were shown to be significantly different (*p* < 0.05). ^1^ HI_0_—group without HI in diet; HI_5_—group with 5% HI in diet; HI_10_—group with 10% HI in diet; HI_15_—group with 15% HI in diet.

**Table 6:** Redox status of the liver of turkeys.

	MDA µmol/kg	GSH + GSSG µmol/kg	CAT U/g Protein	SOD U/g Protein
Group ^1^				
HI_0_	1.107 ^y^	53.56 ^a^	11.92 ^ab^	6.198 ^x^
HI_5_	1.339 ^xy^	50.71 ^a^	10.60 ^b^	5.732 ^xy^
HI_10_	1.631 ^x^	49.96 ^a^	13.95 ^a^	4.785 ^y^
HI_15_	1.367 ^xy^	41.73 ^b^	13.23 ^ab^	5.642 ^xy^
SEM total	0.071	1.200	0.461	0.186
*p*-Value:				
Group	0.072	0.002	0.049	0.054
Linear	0.085	<0.001	0.072	0.108
Quadratic	0.075	0.222	0.739	0.070
Cubic	0.317	0.331	0.031	0.159

^a, b^ mean values within column with unlike superscript letters were shown to be significantly different (*p* < 0.05). ^x, y^ values in same column with no common superscript denote a near significant trend (0.05 < *p* < 0.10). ^1^ HI_0_—group without HI in diet; HI_5_—group with 5% HI in diet; HI_10_—group with 10% HI in diet; HI_15_—group with 15% HI in diet. MDA—malondialdehyde; GSH + GSSG—total glutathione; CAT—catalase; SOD—superoxide dismutase.
